# The population-level economic burden of liver cancer in China, 2019–2030: prevalence-based estimations from a societal perspective

**DOI:** 10.1186/s12962-022-00370-3

**Published:** 2022-07-23

**Authors:** Meng-Di Cao, Cheng-Cheng Liu, Hong Wang, Lin Lei, Maomao Cao, Yuting Wang, He Li, Xin-Xin Yan, Yan-Jie Li, Xin Wang, Ji Peng, Chunfeng Qu, Eleonora Feletto, Ju-Fang Shi, Wanqing Chen

**Affiliations:** 1 Office of Cancer Screening, National Cancer Center / National Clinical Research Center for Cancer / Cancer Hospital, Chinese Academy of Medical Sciences and Peking Union Medical College, Beijing, China; 2 Department of Cancer Prevention and Control, Shenzhen Center for Chronic Disease Control, Shenzhen, China; 3 State Key Lab of Molecular Oncology, National Cancer Center / National Clinical Research Center for Cancer / Cancer Hospital, Chinese Academy of Medical Sciences and Peking Union Medical College, Beijing, China; 4 The Daffodil Centre, The University of Sydney, a joint venture with Cancer Council NSW, Sydney, Australia

**Keywords:** Liver cancer, Cost of illness, China, Population-level, Prevalence

## Abstract

**Background:**

Benchmark data on the population-level economic burden are critical to inform policymakers about liver cancer control. However, comprehensive data in China are currently limited.

**Methods:**

A prevalence-based approach from a societal perspective was used to quantify the annual economic burden of liver cancer in China from 2019 to 2030. Detailed per-case data on medical/non-medical expenditure and work-loss days were extracted from a multicenter survey. The numbers/rates of new/prevalent cases and deaths, survival, and population-related parameters were extracted from the Global Burden of Disease 2019 and the literature. All expenditure data were reported in both 2019 Chinese Yuan (CNY) and United States dollar (US$, for main estimations).

**Result:**

The overall economic burden of liver cancer was estimated at CNY76.7/US$11.1 billion in China in 2019 (0.047% of the local GDP). The direct expenditure was CNY21.6/US$3.1 billion, including CNY19.7/US$2.9 billion for medical expenditure and CNY1.9/US$0.3 billion for non-medical expenditure. The indirect cost was CNY55.1/US$8.0 billion (71.8% of the overall burden), including CNY3.0/US$0.4 billion due to disability and CNY52.0/US$7.5 billion due to premature death. The total burden would increase to CNY84.2/US$12.2 billion, CNY141.7/US$20.5 billion, and CNY234.3/US$34.0 billion in 2020, 2025, and 2030, accounting for 0.102%, 0.138%, and 0.192% of China's GDP, respectively. However, if China achieves the goals of Healthy China 2030 or the United Nations' Sustainable Development Goals for non-communicable diseases, the burden in 2030 would be < CNY144.4/US$20.9 billion.

**Conclusions:**

The population-level economic burden of liver cancer in China is currently substantial and will consistently increase in the future. Sustainable efforts in primary and secondary interventions for liver cancer need to be further strengthened.

**Supplementary Information:**

The online version contains supplementary material available at 10.1186/s12962-022-00370-3.

## Introduction

Liver cancer is a major burdensome cancer worldwide, and China contributes more than half the global burden of liver cancer [1]. Effective interventions have been established in the past two decades in China, including universal neonatal hepatitis B virus (HBV) vaccination, regional dietary aflatoxin control, and appropriate regulations to address primary prevention as well as secondary prevention measures such as screening and early detection [1]. Although this has resulted in positive changes such as a decline in HBV infection over time, the current burden of liver cancer in China is still significant [2]. The 5-year survival rate of liver cancer was much lower than the average of all cancers in 2012–2015 (12.1% vs. 40.5%) [3].

Liver cancer also imposes a substantial economic burden. A multicenter survey (n = 12,342) reported that the per-case average medical expenditure for liver cancer diagnosis and treatment in China doubled from 2002 to 2011 [4]. An earlier systematic review focused on the economic burden of liver cancer in China from 1996 to 2015; among the 32 studies included, only two were conducted at the population level (one at the provincial level and the other at the municipal level) [5]. In an updated review published in 2020 [2], an additional population-level economic burden analysis was included; this is the only national study, to our knowledge, to provide cost data for liver cancer at the national level. In this broad expenditure analysis of hospital care for all cancers in China, which used a Chinese hospital information database, the direct medical cost of liver cancer in 2015 was estimated at CNY8.1 billion [6]; however, the study did not present costs due to disability and premature death for liver cancer.

Because liver cancer presents a significant burden, it must be addressed through evidence-based interventions to achieve the United Nations’ Sustainable Development Goals (SDG) 2030 [7] of reducing premature mortality from non-communicable diseases by one-third and the Chinese goal of increasing the 5-year cancer survival rate by 15% by 2030 [8]. Detailed baseline data on the population-level economic burden of liver cancer are essential in providing a benchmark for future interventions and for evaluating and monitoring primary and secondary liver cancer interventions in China for these goals. Thus, the current study aimed to quantify the comprehensive economic burden of liver cancer in China from 2019 to 2030 at the population level. A prevalence-based approach was previously used to evaluate the population-level economic burden of lung cancer [9, 10]. This study further developed the methodology and integrated detailed local population-specific parameters for liver cancer. The findings will be informative for future policymaking on liver cancer control and related budget allocation in China.

## Methods

### Study design

**Fig. 1:**
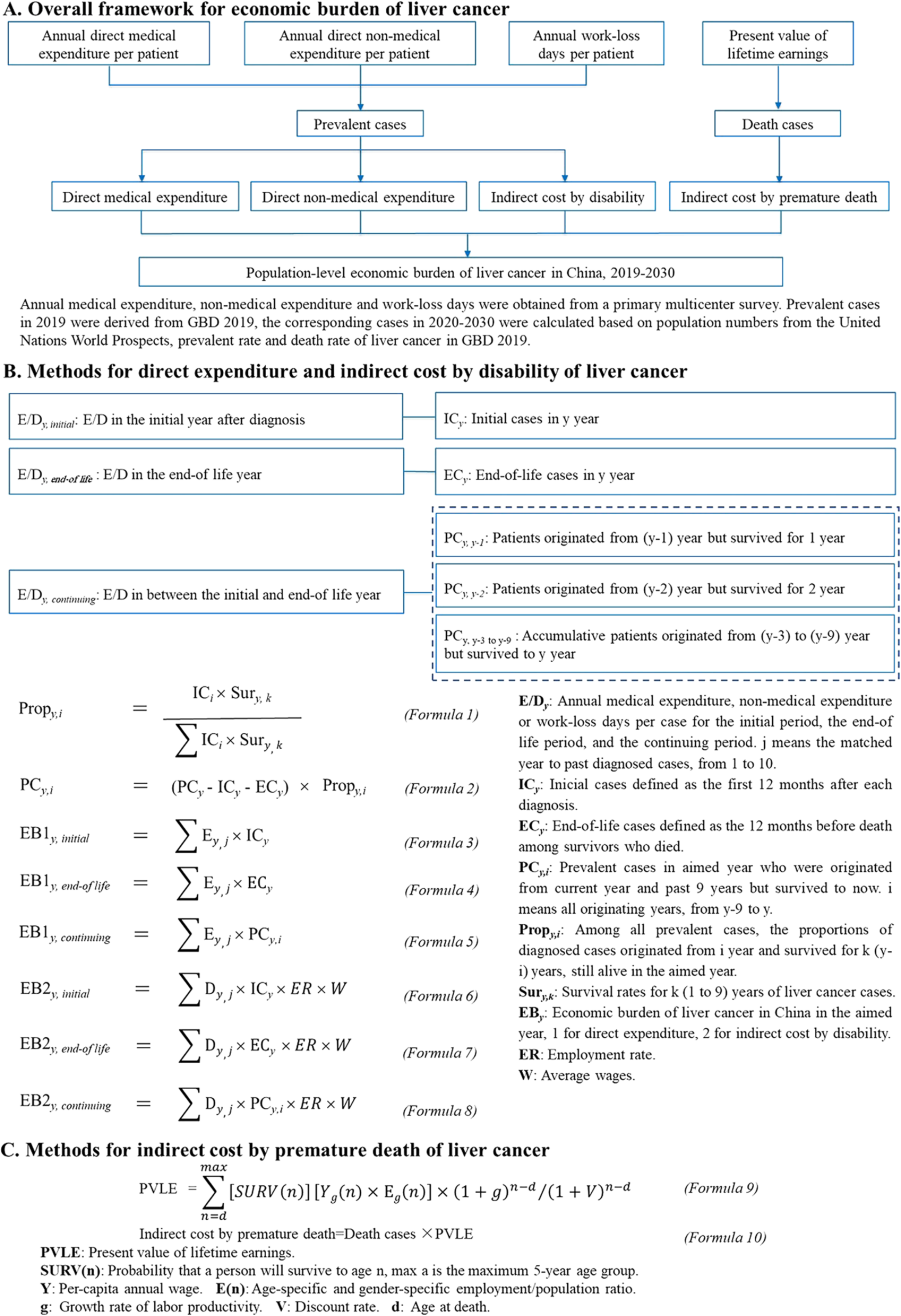
The overall design

A prevalence-based approach from a societal perspective was used to estimate the annual economic burden of liver cancer in China from 2019 to 2030 [9, 10], including direct medical expenditure (DM), direct non-medical expenditure (DNM), indirect cost by disability (IDIS), and indirect cost by premature death (IPD) (Fig. 1A). DM includes overall medical expenditure on liver cancer, while DNM covers expenditures for transportation, additional meals and nutrition, accommodation, hiring informal nursing and other expenditures.

### Data sources

Detailed per-case data on medical expenditure, non-medical expenditure, and work-loss days (for disability-related calculations) were extracted from a hospital-based, multicenter, cross-sectional survey conducted among liver cancer patients in surveyed hospitals covering 13 provinces across China from 2012 to 2014 [11]. A total of 2223 patients were enrolled. Detailed information, including demographics, clinical characteristics, medical and non-medical expenditures and work-loss days by clinical visits, were collected through face-to-face interviews and questionnaires [11].

The disease burden of liver cancer was obtained from the Global Burden of Disease (GBD) 2019 (Additional file 1: Table S1). Age-specific survival rates were taken from the 2015 Life Table for China from the World Health Organization (WHO) [12]. Employment rates from the Organization for Economic Co-operation and Development (OECD) [13] and estimated future population sizes from the United Nations (UN) World Population Prospects [14] were applied (Additional file 1: Table S2). Additionally, to calculate the proportions of prevalent cases in each year, overall survival rates were extracted from reports of national cancer registries [2] and other population-based studies [15].

### Estimations of economic burden

#### Direct expenditure and indirect cost by disability

DM and DNM were estimated by summarizing the products of annual direct expenditure and matched prevalent cases in the corresponding years after diagnosis (Formula 3–5 in Fig. 1B). IDIS was estimated by summarizing the products of annual work-loss days, matched prevalent cases in each year post-diagnosis, employment ratios, and daily wages (Formula 6–8 in Fig. 1B).

Prevalent cases were divided into 10 groups, including newly diagnosed cases in 2019 (y) and cases originating from each previous year (y-1 to y-9) while surviving until 2019 (Formula 1–2 in Fig. 1B). Estimations for DM, DNM and IDIS were also broken down into a 10-year group [16] combined with expenditure patterns [17], which resolved expenditure into three clinically relevant phases: the initial phase (the first 12 months after each diagnosis), the end-of-life (EOL) phase (the 12 months before death among survivors who died) and the continuing phase (the months between the initial and EOL phases). Annual direct expenditure (medical and non-medical) and work-loss days per prevalent liver cancer patient were calculated for each 12-month period after initial diagnosis. To maintain robust estimates for DM, DNM and IDIS, the analyzed sample for each 12-month period was larger than 50, so we used actual cost in the year 1 to year 3 for analysis and converted the year 4 to year 10 expenditure by the expenditure patterns [17]. Thus, the year 1 expenditure was considered the initial phase, the year 2 to year 3 expenditures were used as the original cost data, the year 4 to year 9 expenditures were calculated by the cost in the year 1 multiplied by the corresponding ratio (cost in the continuing phase/cost in the initial phase), and the year 10 expenditure was calculated by the cost in the year 1 multiplied by the corresponding ratio (cost in the continuing phase/cost in the EOL phase). Under this assumption, DM, DNM and IDIS were calculated by Formula 3–8 in Fig. 1B. The annual direct medical expenditure, non-medical expenditure and work-loss days per liver cancer patient in China in 2019 are presented in Additional file 1: Table S3. All expenditure costs were discounted at a rate of 3% together with an annual growth rate of 6.29% [4].

#### Indirect cost by premature death

IPD was estimated for patients aged 15 years old (working age) to life expectancy (male: 74.5; female: 80.0) [18]. The present value of lifetime earnings (PVLE) was estimated via 5-year age groups by the human capital approach [19], and IPD was calculated by Formula 9–10 in Fig. 1C. An annual productivity growth rate of 8.7% (annual change of gross domestic product (GDP) in the last 5 years) [20] was assumed to consider the potential growth of future earnings.

### Prediction of the future economic burden

The future economic burden was projected by a growing disease burden and an annual growth rate of 6.29% per patient expenditure [4]. Disease burden was projected based on population numbers estimated by the medium fertility variant from the UN World Prospects [14] and rates of incidence, prevalence, and mortality from GBD 2019 [18]. We established three scenarios in the projection. (1) In the demographic change scenario, we assumed that changes in aging and urbanization were the only drivers of disease burden. (2) In the base case scenario, the disease burden was further projected based on the current status in 2019 and the rates of changes in past trend. Mean annual change rates in the age- and sex-specific incidence, prevalence and mortality in China were estimated using 2009–2019 data from GBD 2019. (3) In the target scenario, we explored the predicted economic burden in 2030 under the realization of the ‘SDG 2030’ or ‘Healthy China 2030’ goals.

### Sensitivity analysis

We conducted one-way sensitivity analysis, scenario and probabilistic sensitivity analyses to explore uncertainty in the economic burden from 2019 to 2030. The differences in costs-per-case were vital for economic burden assessments. For DME and DNME, our per-case data did not include the lower-level hospital, which could lead to an overestimation, and the survey was adopted before discharge, which could exclude some expenditures that occurred subsequently. Therefore, we assessed the variation in the estimated total cost resulting from a 30% change in the annual direct medical expenditure and non-medical expenditure per patient. An annual growth rate of 4.85% (lower) and 8% (higher) for direct expenditure, observed in a multicenter survey on the economic burden of liver cancer [4], were input to test the impact. For IPD, we considered different growing productivity and distinct ranges of working age. An annual growth rate of 6.7% was applied as a conservative estimation based on OECD estimates of GDP from 2014 to 2019 [20]. The worst productivity/earnings growth rate of − 6.8% was assumed by taking the economic situation during the coronavirus disease 2019 (COVID-19) pandemic into account based on GDP in the first quarter in China [22]. Distinct ranges of working age included 15–60 for males and 15–55 for females as required by China [23], 15–64 defined by the OECD [13] and 30–69 as recommended by a global estimation [24]. In our base-case analysis, we assumed that all liver cancer patients received treatment; however, information from the 5th National Health Service Survey indicated that the consultation rate was only 62% [21]. In addition, the effect of discounting on estimation was assessed using the rate of 5%. Data from Cancer Tomorrow in GLOBOCAN 2020 [25] were used to evaluate the potential influence of the data source.

Based on all the variables in the one-way sensitivity analysis, the best and worst scenarios are considered to test the extreme situation by combining the different assumption changes. In the best scenario, the overall changes of all parameters that can reduce economic burden are considered and the worst scenario is considered conversely. A probabilistic sensitivity analysis, based on 5000 simulations, was performed on key variables that could be sampled randomly obtained from baseline data sources ‘annual direct medical expenditure’, ‘annual direct non-medical expenditure’, and ‘work-loss days’ per liver cancer patient in China in 2019, to test the robustness of the results and define the proper 95% Confidence Interval (CI).

### Statistical analysis

The analyses of estimations and predictions of economic burden were conducted with SAS version 9.4 software (SAS Institute Inc., Cary, USA) and Microsoft Excel 2019. All expenditure data were reported in 2019 Chinese Yuan (CNY) and converted to 2019 United States dollar (US$) in the main estimations (exchange rate: 1US$ = 6.8985 CNY).

## Results

### Economic burden in 2019

**Fig. 2:**
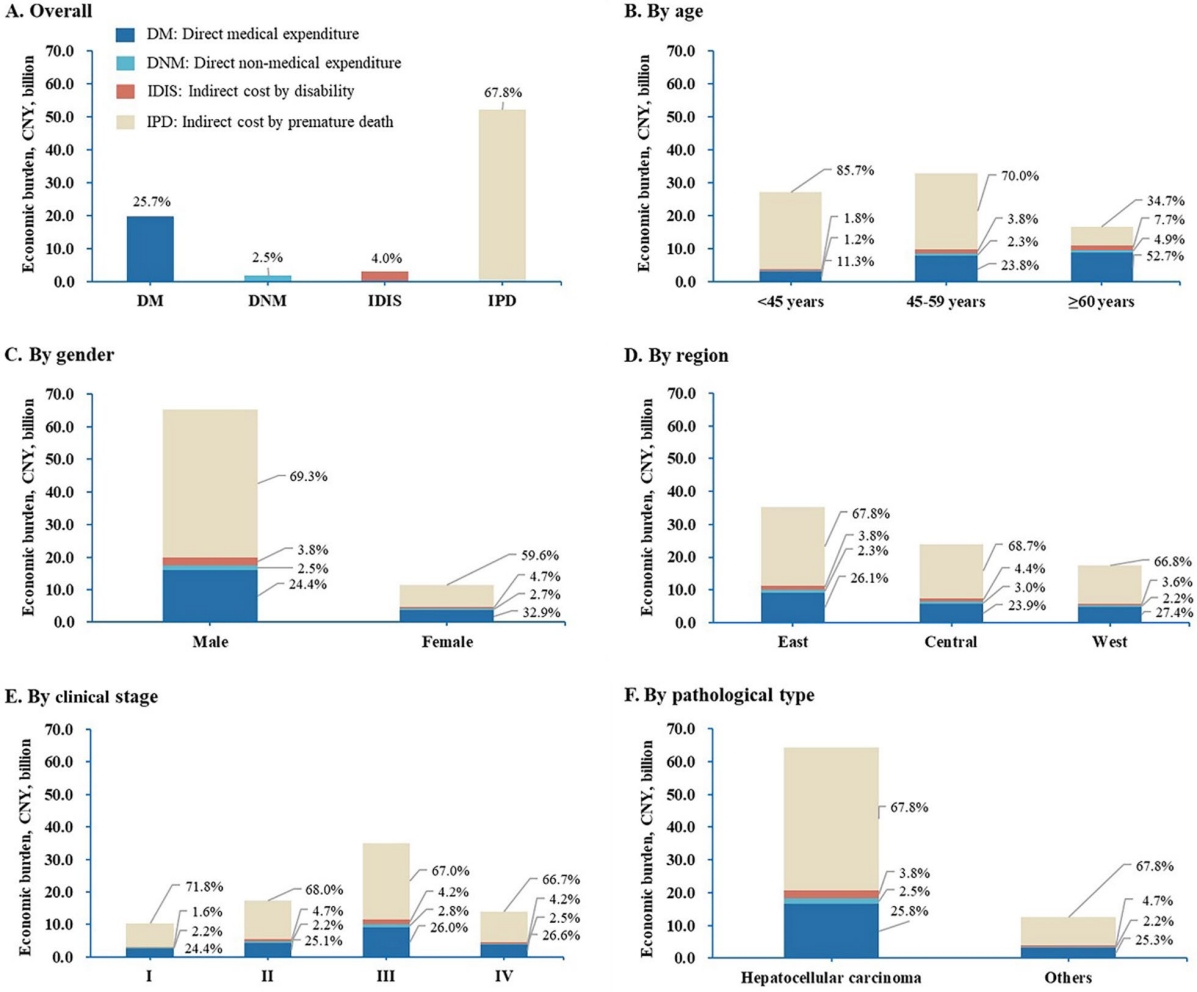
The breakdowns of the population-level economic burden of liver cancer in China in 2019, the overall and by subgroup

The total estimated economic burden of liver cancer in 2019 was CNY76.7 (US$11.1) billion, equivalent to 0.047% of the OECD-reported GDP [20] of China in 2019 (US$23,601.4 billion). If we adopted locally generated GDP (CNY90,031.0 billion) [26], the share of GDP would be 0.085%. The total direct expenditure was CNY21.6 (US$3.1) billion. In this expenditure, DM was CNY19.7 (US$2.9) billion, approximately 0.334% of the total healthcare expenditure (THE) [27] in China in 2019 (Table 1). The total indirect cost was CNY55.1 (US$8.0) billion, accounting for 71.8% of the total economic burden. Among the indirect costs, 94.5% resulted from IPD (CNY52.0/US$7.5 billion), and the IDIS was CNY3.0 (US$0.4) billion (Fig. 2).

**Table 1:** Estimated population-level economic burden of liver cancer in China in 2019, overall and by subgroups

Overall and subgroups	Direct expenditure				Indirect cost			Total economic burden		
									GDP^b^, %	
	Medical, CNY billion	Non-medical, CNY billion	Sub-total, CNY billion	THE^a^, %	Disability, CNY billion	Premature death, CNY billion	Sub-total, CNY billion	Total, CNY billion	China^c^	OECD^d^
Overall	19.7 (US$2.9 billion)	1.9 (US$0.3 billion)	21.6 (US$3.1 billion)	0.366	3.0 (US$0.4 billion)	52.0 (US$7.5 billion)	55.1 (US$8.0 billion)	76.7 (US$11.1 billion)	0.085	0.047
Age at diagnosis, years										
< 45	3.0	0.3	3.4	0.057	0.5	23.2	23.7	27.0	0.030	0.017
45–59	7.8	0.8	8.6	0.145	1.3	23.1	24.3	32.9	0.037	0.020
≥ 60	8.8	0.8	9.7	0.163	1.3	5.8	7.1	16.8	0.019	0.010
Gender										
Male	15.9	1.6	17.5	0.296	2.5	45.2	47.7	65.2	0.072	0.040
Female	3.8	0.3	4.1	0.070	0.5	6.9	7.4	11.5	0.013	0.007
Region										
East	9.2	0.8	10.0	0.170	1.3	23.9	25.3	35.3	0.039	0.022
Central	5.7	0.7	6.4	0.109	1.1	16.4	17.5	23.9	0.027	0.015
West	4.8	0.4	5.2	0.088	0.6	11.7	12.3	17.5	0.019	0.011
Clinical stage										
I	2.5	0.2	2.7	0.046	0.2	7.4	7.6	10.3	0.011	0.006
II	4.4	0.4	4.7	0.080	0.8	11.8	12.6	17.3	0.019	0.011
III	9.1	1.0	10.1	0.171	1.5	23.5	25.0	35.1	0.039	0.022
IV	3.7	0.3	4.1	0.069	0.6	9.3	9.9	14.0	0.016	0.009
Health insurance										
UEBMI	8.2	0.8	9.0	0.153	1.3	20.5	21.8	30.8	0.034	0.019
URBMI	3.6	0.3	3.9	0.067	0.5	9.1	9.6	13.5	0.015	0.008
NRCMS	7.4	0.7	8.1	0.137	1.2	20.5	21.7	29.8	0.033	0.018
Commercial insurance	0.1	0.0	0.1	0.001	0.0	0.5	0.5	0.6	0.001	0.000
Self-pay	0.2	0.0	0.2	0.004	0.0	1.0	1.0	1.2	0.001	0.001
Others	0.2	0.0	0.2	0.004	0.0	0.5	0.6	0.8	0.001	0.000
Pathological type										
Hepatocellular carcinoma	16.6	1.6	18.2	0.308	2.4	43.6	46.0	64.3	0.071	0.039
Other	3.1	0.3	3.4	0.058	0.6	8.4	9.0	12.5	0.014	0.008

^a^Total health expenditure in China, 2019 [25]. ^b^Gross domestic product in China, 2019. ^c^GDP  =  CNY90,030.95 billion from China Statistical Year Book 2019 [26]. ^d^GDP = US$23,601.366 billion from OECD (Organization for Economic Co-operation and Development) [20]. UEBMI: urban employee basic medical insurance; URBMI: urban resident basic medical insurance; NRCMS: new rural cooperative medical system

The cost of liver cancer varies widely across demographic and tumor characteristics. Patients aged 45 to 59 years (42.9%), who are male (85.0%), in the eastern region (46.0%), with stage III disease (45.7%), and who have urban employee basic medical insurance (40.2%) and hepatocellular carcinoma (83.8%) represent the largest proportion of the economic burden. The distribution patterns in the subgroups for DM, DNM, IDIS and IPD were similar to the total burden (Table 1 and Fig. 2).

### Economic burden in the future

**Fig. 3:**
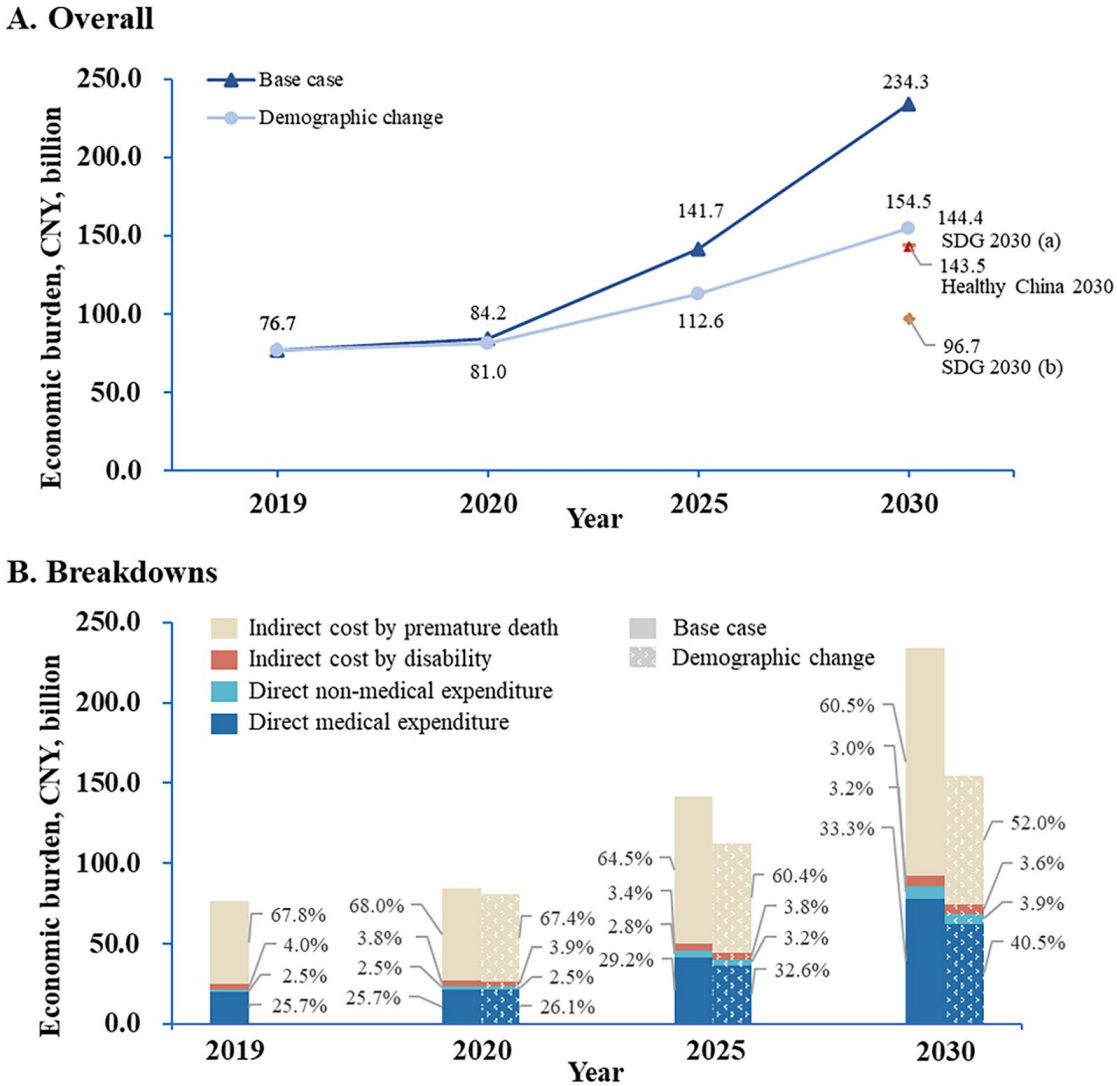
The overall burden and breakdown of the estimated population-level economic burden of liver cancer in China, 2019–2030. Demographic change scenario: only considered the demographic changes. Base case scenario: simultaneously considered the changes in demography and the increasing disease burden of liver cancer in China (based on the GBD 2019 data on incidence, mortality and prevalence 2009–2019). SDG 2030: one of the Sustainable Development Goals proposed by the United Nations, which is, by 2030, to reduce by one third premature mortality from non-communicable diseases through prevention and treatment and promote mental health and well-being; the sub-scenario (**a**) considered the demographic changes and the reduction in liver cancer mortality; the sub-scenario (**b**) furtherly considered the reductions in incidence and prevalence of liver cancer. Healthy China 2030 scenario: by 2030, to achieve a 15% increase in 5-year survival rate for cancer

In the base case scenario, the economic costs of liver cancer in China for 2020, 2025, and 2030 were CNY84.2 (US$12.2) billion, CNY141.7 (US$20.5) billion, and CNY234.3 (US$34.0) billion, representing up to 0.102%, 0.138%, and 0.192% of China’s GDP (GDP long-term forecast from OECD) [20], respectively. Compared with 2019, these numbers would increase by 9.8%, 84.8%, and 205.5% in 2020, 2025 and 2030, respectively. In contrast, if demographic changes were the only drivers, the economic burden would be CNY81.0 (US$11.8) billion (2020), CNY112.6 (US$16.3) billion (2025), and CNY154.5 (US$22.4) billion (2030) (Fig. 3A).

However, if China achieved the SDG goal by averting only premature death from liver cancer together with a similar reduction in the prevalence and incidence rates, the economic burden of liver cancer in 2030 would decrease to CNY96.7 (US$14.0) billion (approximately 0.264% of China’s GDP) with a reduction of 58.7% relative to the base case scenario in 2030. Even if the reduction in premature mortality did not impact prevalence and incidence rates, the economic burden would decrease by 38.4% (CNY144.4, US$20.9 billion). Additionally, if the ‘Healthy China 2030’ goal was achieved, it would be CNY143.5 (US$20.8) billion, which is 38.7% lower than the base case scenario for 2030 (Fig. 3A).

Furthermore, the breakdowns of the estimated economic burden of liver cancer show that the proportion of indirect costs (IDIS and IPD) in the total economic burden will decline over time; in contrast, DM and DNM will increase. In the demographic change scenario, the variation in economic costs is even more pronounced (Fig. 3B).

### Sensitivity analysis

**Fig. 4:**
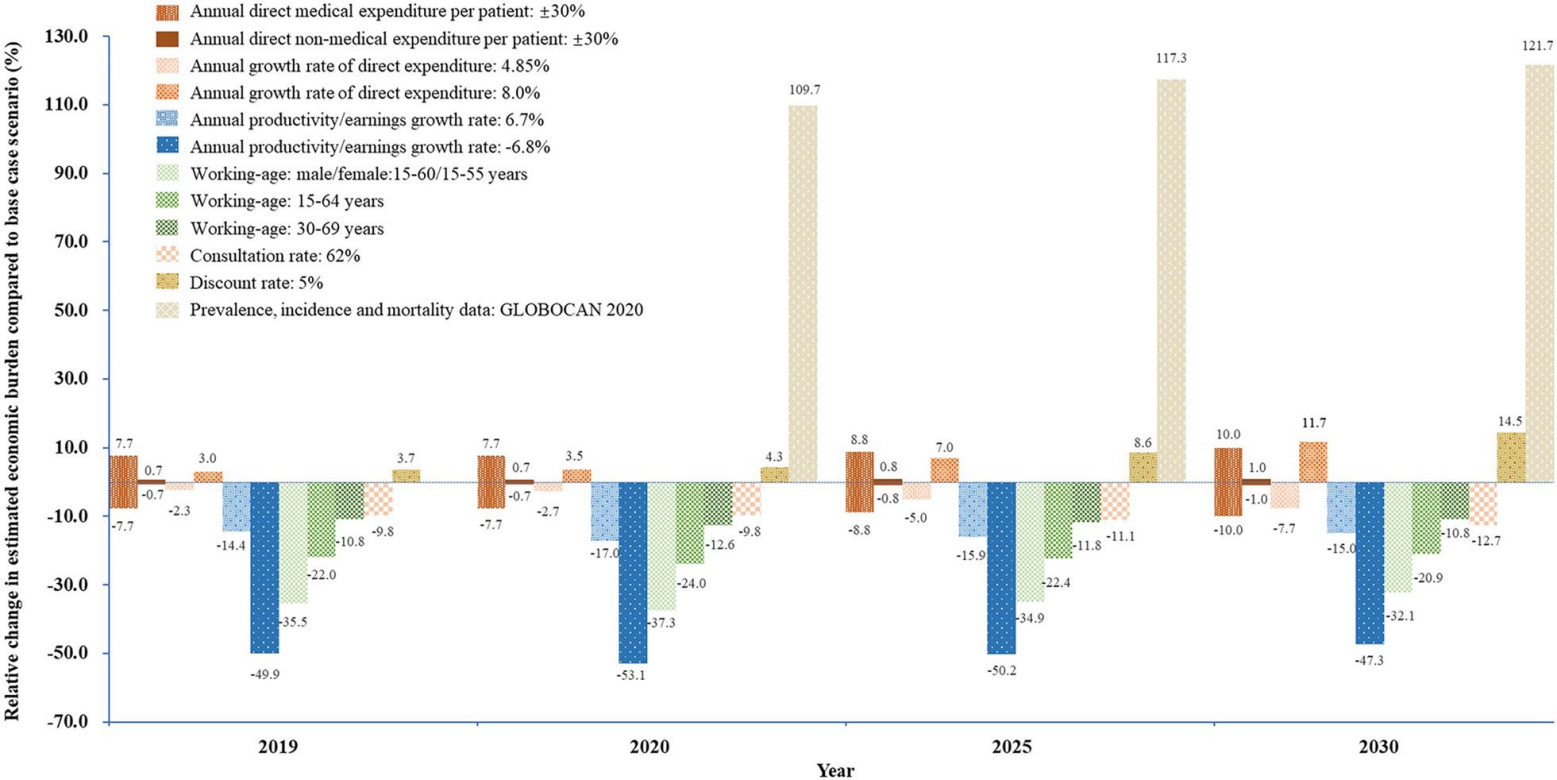
Sensitivity analyses for the estimated population-level economic burden of liver cancer in China, 2019–2030

In the one-way sensitivity analysis, most changes in variables will reduce the economic burden, between 0.7% (annual direct medical expenditure per patient: − 30%) to 49.9% (annual productivity growth rate: − 6.8%) in 2019. If medical expenditure rises at a lower (4.85%) annual rate, the economic burden decreases by 2.3% in 2019; in contrast, it grows by 3.0% if medical expenditure rises at a faster (8.0%) annual rate. Considering a lower consultation rate, the overall economic burden in 2019 decreased by 9.8% (Fig. 4).

For annual per-case data, the variation in the changes in the annual direct medical expenditure per patient (± 7.7% ~  ± 10.0%) was much more obvious than that of non-medical expenditure (± 0.7% ~  ± 1.0%) from 2019 to 2030. Moreover, the variation of the annual productivity growth rate and working-age range plays an important role in the estimations, which may greatly reduce the long-term economic burden. In the estimation of the 5% discount rate, 3.7% ~ 14.5% increases in economic burden were observed. In addition, the economic burden rose wildly from 109.7% (2020) to 121.7% (2030) when we used the disease burden of liver cancer from GLOBOCAN 2020 (Fig. 4).

In the worst scenario, the economic burden could be CNY90.3 billion in 2019, which is 17.9% higher than the base case. More obviously, the economic burden could be CNY19.5 billion in 2019 in the best scenario, which is 74.5% lower than the base case. The probabilistic sensitivity analysis resulted in an average cost for the direct medical expenditure of CNY22.280 billion (95% CI: CNY22.259 ~ CNY22.302 billion), for the direct non-medical expenditure of CNY2.159 billion (95% CI: CNY2.156 ~ CNY2.163 billion) and for the indirect costs of CNY3.039 billion (95% CI: CNY3.036 ~ CNY3.043 billion). The total economic burden in 2019 would be estimated at CNY79.516 billion (95% CI: CNY79.487 ~ CNY79.545 billion).

## Discussion

To our knowledge, this is the most comprehensive and detailed analysis of the population-level economic burden of liver cancer conducted in China. This study provides benchmark data on the population-level economic burden, which is essential to inform policymaking in liver cancer control and related budget allocation. Using a prevalence-based approach and integrating multisource data, this study found that the economic burden of liver cancer in China was substantial in 2019 (CNY76.7/US$11.1 billion, of which 71.8% were indirect costs, accounting for 0.047% of China’s GDP) and would continue to increase (CNY234.3/US$34.0 billion in 2030). However, the burden in 2030 would be <CNY144.4/US$20.9 billion if China achieved the ‘Healthy China 2030’ goal or the SDG.

The proportion of the economic burden of one disease to the local GDP (GDP percent) is used as an indicator to compare different economies. Our study suggests that China’s GDP proportion attributable to cancer is lower than that of Japan (0.102%, 607.2 billion Japanese Yen in 2014) [28] and Korea (0.117%, US$2.27 billion in 2015) [29], based on the available literature. For other Western countries, such as the US, where the main causes of liver cancer are quite different from Asia [30], the overall financial cost per hospitalization for liver cancer was US$59,465 in 2011 [31], and a recent systematic review showed no other studies of the population-level economic burden of liver cancer in the US to date [32]. As expected, the GDP percent for liver cancer (0.085%) is lower than that of lung cancer (0.205%). The main reason may be the differences in disease burden.

Our study included direct non-medical expenditure and indirect costs, which are rarely reported in existing studies on the economic burden of China’s population, in addition to direct medical expenditure. Using large-scale inpatient medical records and assuming ratios of outpatient payments to inpatient payments, a previous study reported a national expenditure of hospital care as CNY8.1 billion for liver cancer [6], which is ~ 2/5 of the direct medical expenditure in our study. The differences are likely due to the different data sources of per-case expenditures, which were greater from tertiary hospitals in our study than from tertiary or lower-level hospitals in the study by Cai et al. [6] Our analysis also suggests a high proportion of indirect cost in the total burden for liver cancer (71.8%), which is higher than that of lung cancer (55.7%) [10]. The difference is probably due to the relatively lower survival rate of liver cancer (12.1%) [3].

The findings suggest that the overall economic burden of liver cancer in China will continue to increase (CNY234.3 billion in 2030), but the extent of the increase could be largely delayed if appropriate interventions are implemented in China. Population aging, continuing urbanization, and increasing liver cancer burden contribute to the increase in the projected economic burden of liver cancer over the next decade in China. However, if the goals of SDG 2030 (a), SDG 2030 (b) and Healthy China 2030 can be achieved through application of policy approaches and interventions, the economic burden would decline by 38.4%, 58.7% and 38.7%, respectively. These reductions highlight the need for actions to reduce the economic burden by aiming to reduce mortality and increase the survival rate of liver cancer. These actions require further advances in clinical treatment, primary prevention (e.g. various HBV vaccination interventions and antiviral therapy) and secondary prevention (e.g. liver cancer screening in high-risk populations, early detection or surveillance). The shifting burden from indirect cost (IDIS and IPD) to direct expenditure (DM and DNM) in the breakdown of the estimated economic burden indicates that due to the aging population and urbanization, the survival period of patients is prolonged, and the number of patients and the corresponding cost increase. In Japan [28], decreased IPD was the primary factor contributing to the total economic burden, mainly due to the decreased fatality rate and increased age at death. Therefore, the urgent need to formulate and implement relevant control measures is growing because appropriate interventions could weaken this dynamic growth trend and reduce the economic burden.

In the sensitivity analysis, a change in the annual productivity/earnings growth rate has a greater influence on the overall economic burden than a change in the annual growth rate of direct expenditure because it mainly affected IPD, which accounts for the largest proportion of the overall burden. Regarding the impact of working age, when assuming shortened age ranges of three types (in which the maximum age limit is 69 rather than below the sex-specific life expectancy), the overall burden would be considerably reduced. The overall burden was found to be most sensitive (doubled) to data sources of the disease burden when using a set of parameters from GLOBOCAN, in which higher incidence and mortality estimates were adopted. Considering variable trends in the pathogenic factors of liver cancer (HBV, hepatitis C virus, and non-virus-related factors), our future trend estimation, which used future disease burden data from the GBD website, reflects the changes in etiology to a certain extent.

The current analysis can be regarded as a preliminary estimate of the economic burden at the population level, but it has limitations. First, the precision of the current estimate relies on the per-case data from a previous hospital-based survey that involved individual-level and hospital-level (high-level) selection bias, which may cause an overestimation of the medical economic burden; however, this is the best publicly available data to date. Second, when patients were diagnosed at very late stages, some refused treatment. The extent remains unknown, and we used a simplified estimation based on the consultation rate. Furthermore, the treatment costs incurred outside hospitals, such as drugs purchased in retail pharmacies, cannot be accessed and considered in medical expenditures. Both estimations affect medical expenditure and indirect costs. Third, the current combination of expenditure and cases was not well matched. If death occurred within 1 year after diagnosis, the cost was regarded as the EOL phase according to the definition, which we did not consider this situation. Finally, this study is based on a variety of assumptions and databases, and other uncertainties may exist.

## Conclusions

The population-level economic burden of liver cancer in China in 2019 is substantial and will consistently increase in the next decade. To delay or even reverse this increasing trend, sustainable efforts in primary and secondary interventions need to be further strengthened in China.

## Supplementary Information

**Additional file 1: Table S1.** Inputting parameters of disease burden of liver cancer in China, 2019. **Table S2.** Inputting parameters of survival probability, employment rates and numbers population in China. **Table S3.** Annual direct medical, non-medical expenditure and work-loss days per liver cancer patient in China in 2019 by year post-diagnosis.

## Data Availability

The datasets generated and/or analyzed during the current study are not publicly available but are available from the corresponding author on reasonable request.

## Abbreviations

GBD: Global Burden of Disease Study
CNY: Chinese Yuan
GDP: Gross domestic product
SDG: Sustainable development goals
HBV: Hepatitis B virus
DM: Direct medical expenditure
DNM: Direct non-medical expenditure
IDIS: Indirect cost by disability
IPD: Indirect cost by premature death
WHO: World Health Organization
OECD: Organization for Economic Co-operation and Development
UN: United Nations
PVLE: Present value of lifetime earnings
THE: Total healthcare expenditure
US: United States
EOL: End-of-life
UEBMI: Urban employee basic medical insurance
URBMI: Urban resident basic medical insurance
NRCMS: New rural cooperative medical system
COVID-19: Coronavirus disease 2019
CI: Confidence interval
