# Supplementary material for: The population-level economic burden of liver cancer in China, 2019–2030: prevalence-based estimations from a societal perspective
Source: Cost Eff Resour Alloc. 2022 Jul 23;20:36. doi: 10.1186/s12962-022-00370-3 (PMC9308023; doi:10.1186/s12962-022-00370-3)
Supplement: Supplementary file 1 — Additional file 1: Table S1. Inputting parameters of disease burden of liver cancer in China, 2019. Table S2. Inputting parameters of survival probability, employment rates and numbers population in China. Table S3. Annual direct medical, non-medical expenditure and work-loss days per liver cancer patient in China in 2019 by year post-diagnosis. [file 12962_2022_370_MOESM1_ESM.docx]

**Manuscript title:** The population-level economic burden of liver cancer in China, 2019-2030: prevalence-based estimations from a societal perspective

**Additional file 1**

**Table S1 Inputting parameters of** **disease burden of liver cancer in China, 2019**

**Table S2 Inputting parameters of survival probability, employment rates and numbers population in China**

**Table S3 Annual direct medical, non-medical expenditure and work-loss days per liver cancer patient in China in 2019 by year post-diagnosis**

**Table S1 Inputting parameters of disease burden of liver cancer in China, 2019**

| **Characteristics** | | **No. of liver cancer cases** | | | | | | | |  | **Rates, per 100 000 population** | | | | | | | |
| --- | --- | --- | --- | --- | --- | --- | --- | --- | --- | --- | --- | --- | --- | --- | --- | --- | --- | --- |
|  |  | **Incidence** | |  | **Death** | |  | **Prevalence** | |  | **Incidence** | |  | **Death** | |  | **Prevalence** | |
|  |  | **Male** | **Female** |  | **Male** | **Female** |  | **Male** | **Female** |  | **Male** | **Female** |  | **Male** | **Female** |  | **Male** | **Female** |
| Total | | 159 787 | 50 675 |  | 139 026 | 48 674 |  | 228 332 | 62 041 |  | 22.05 | 7.26 |  | 19.18 | 6.98 |  | 31.50 | 8.89 |
| By age, years | | | | | | | | | | | | | | | | | | |
|  | <1 | 46 | 37 |  | 57 | 39 |  | 99 | 84 |  | 0.56 | 0.53 |  | 0.70 | 0.56 |  | 1.22 | 1.21 |
|  | 1-4 | 42 | 54 |  | 68 | 54 |  | 92 | 125 |  | 0.12 | 0.17 |  | 0.19 | 0.18 |  | 0.26 | 0.41 |
|  | 5-9 | 74 | 60 |  | 35 | 29 |  | 85 | 87 |  | 0.19 | 0.18 |  | 0.09 | 0.09 |  | 0.22 | 0.26 |
|  | 10-14 | 119 | 73 |  | 54 | 33 |  | 192 | 116 |  | 0.31 | 0.23 |  | 0.14 | 0.10 |  | 0.50 | 0.36 |
|  | 15-19 | 174 | 65 |  | 94 | 34 |  | 441 | 181 |  | 0.43 | 0.18 |  | 0.24 | 0.10 |  | 1.10 | 0.52 |
|  | 20-24 | 495 | 158 |  | 295 | 91 |  | 1 057 | 380 |  | 1.16 | 0.40 |  | 0.69 | 0.23 |  | 2.47 | 0.97 |
|  | 25-29 | 1 385 | 287 |  | 890 | 179 |  | 3 265 | 646 |  | 2.46 | 0.53 |  | 1.58 | 0.33 |  | 5.79 | 1.19 |
|  | 30-34 | 3 751 | 586 |  | 2 697 | 407 |  | 6 724 | 1 105 |  | 5.74 | 0.92 |  | 4.13 | 0.64 |  | 10.30 | 1.73 |
|  | 35-39 | 5 935 | 830 |  | 4 444 | 605 |  | 10 219 | 1 477 |  | 11.55 | 1.68 |  | 8.65 | 1.22 |  | 19.88 | 2.98 |
|  | 40-44 | 10 505 | 1 473 |  | 8 024 | 1 119 |  | 18 001 | 2 570 |  | 20.25 | 2.96 |  | 15.47 | 2.25 |  | 34.70 | 5.16 |
|  | 45-49 | 16 205 | 2 552 |  | 12 792 | 2 066 |  | 26 701 | 3 917 |  | 26.21 | 4.29 |  | 20.69 | 3.47 |  | 43.18 | 6.58 |
|  | 50-54 | 21 446 | 4 321 |  | 17 287 | 3 593 |  | 33 531 | 6 307 |  | 34.13 | 6.94 |  | 27.51 | 5.77 |  | 53.36 | 10.13 |
|  | 55-59 | 20 480 | 5 306 |  | 17 044 | 4 564 |  | 30 650 | 7 323 |  | 42.99 | 11.24 |  | 35.77 | 9.67 |  | 64.33 | 15.52 |
|  | 60-64 | 20 289 | 6 366 |  | 17 418 | 5 699 |  | 29 013 | 8 231 |  | 51.41 | 16.29 |  | 44.14 | 14.58 |  | 73.52 | 21.06 |
|  | 65-69 | 20 894 | 8 120 |  | 19 097 | 7 751 |  | 27 299 | 9 418 |  | 60.48 | 22.66 |  | 55.28 | 21.63 |  | 79.02 | 26.28 |
|  | 70-74 | 16 172 | 7 449 |  | 15 488 | 7 511 |  | 18 584 | 8 224 |  | 69.32 | 30.37 |  | 66.39 | 30.62 |  | 79.66 | 33.53 |
|  | 75-79 | 10 914 | 5 508 |  | 11 214 | 6 009 |  | 11 886 | 5 480 |  | 77.20 | 35.06 |  | 79.33 | 38.25 |  | 84.08 | 34.88 |
|  | 80-84 | 6 989 | 4 262 |  | 7 514 | 4 852 |  | 6 950 | 3 938 |  | 83.45 | 39.86 |  | 89.72 | 45.38 |  | 82.98 | 36.83 |
|  | 85-89 | 3 441 | 2 340 |  | 3 897 | 2 802 |  | 3 268 | 1 932 |  | 115.56 | 42.34 |  | 130.87 | 50.70 |  | 109.74 | 34.96 |
|  | 90-94 | 375 | 675 |  | 525 | 970 |  | 247 | 421 |  | 97.01 | 36.34 |  | 135.96 | 52.24 |  | 63.82 | 22.67 |
|  | 95+ | 57 | 156 |  | 92 | 270 |  | 29 | 79 |  | 158.90 | 37.90 |  | 253.66 | 65.69 |  | 80.94 | 19.20 |

Notes: Total and age/gender-specific incident, death and prevalent liver cancer patients were obtained from the Global Burden of Disease 2019.^1^ Detailed cases specific to other characteristics were based on total cases from GBD 2019 and relative proportions from the hospital-based, multicenter and cross-sectional survey.^2^

**Table S2 Inputting parameters of survival probability, employment rates and numbers population in China**

| **Characteristics** | | **Survival probability** | |  | **Employment rates, %** | |  | **No. of population (in thousands)** | | | | | | | |
| --- | --- | --- | --- | --- | --- | --- | --- | --- | --- | --- | --- | --- | --- | --- | --- |
|  |  |  |  |  |  |  |  | **2020** | |  | **2025** | |  | **2030** | |
|  |  | **Male** | **Female** |  | **Male** | **Female** |  | **Male** | **Female** |  | **Male** | **Female** |  | **Male** | **Female** |
| Total | | NA | NA |  | 76.1 | 61.7 |  | 738 247 | 701 076 |  | 746 461 | 711 448 |  | 748 159 | 716 181 |
| By age，years | |  |  |  |  |  |  |  |  |  |  |  |  |  |  |
|  | 0-4 | 0-1: 0.992 1-4: 0.999 | 0-1: 0.992 1-4: 0.999 |  | NA | NA |  | 44 456 | 39 476 |  | 40 108 | 36 234 |  | 37 007 | 34 026 |
|  | 5-9 | 0.998 | 0.998 |  | NA | NA |  | 46 320 | 40 415 |  | 44 355 | 39 399 |  | 40 025 | 36 168 |
|  | 10-14 | 0.999 | 0.999 |  | NA | NA |  | 45 350 | 38 913 |  | 46 239 | 40 354 |  | 44 285 | 39 344 |
|  | 15-19 | 0.998 | 0.998 |  | 32.1 | 29.7 |  | 44 103 | 38 239 |  | 45 188 | 38 776 |  | 46 088 | 40 224 |
|  | 20-24 | 0.997 | 0.998 |  | 71.8 | 64.9 |  | 46 274 | 40 884 |  | 43 827 | 38 000 |  | 44 927 | 38 551 |
|  | 25-29 | 0.996 | 0.997 |  | 92.8 | 78.7 |  | 51 523 | 46 466 |  | 45 953 | 40 629 |  | 43 534 | 37 765 |
|  | 30-34 | 0.995 | 0.997 |  | 95 | 80.6 |  | 66 443 | 62 296 |  | 51 178 | 46 215 |  | 45 654 | 40 406 |
|  | 35-39 | 0.994 | 0.996 |  | 95 | 81.9 |  | 51 346 | 48 746 |  | 66 016 | 62 005 |  | 50 855 | 45 991 |
|  | 40-44 | 0.992 | 0.994 |  | 94.7 | 82.6 |  | 49 289 | 46 985 |  | 50 939 | 48 468 |  | 65 550 | 61 689 |
|  | 45-49 | 0.988 | 0.991 |  | 93.1 | 78 |  | 61 173 | 58 664 |  | 48 770 | 46 642 |  | 50 444 | 48 137 |
|  | 50-54 | 0.979 | 0.985 |  | 87.8 | 61.5 |  | 62 348 | 61 097 |  | 60 195 | 58 060 |  | 48 042 | 46 187 |
|  | 55-59 | 0.965 | 0.975 |  | 79 | 53.3 |  | 49 958 | 48 782 |  | 60 710 | 60 088 |  | 58 727 | 57 164 |
|  | 60-64 | 0.934 | 0.953 |  | 57.8 | 40.3 |  | 38 917 | 38 597 |  | 47 639 | 47 353 |  | 58 105 | 58 458 |
|  | 65-69 | 0.882 | 0.915 |  | 44.5 | 27.4 |  | 36 527 | 37 623 |  | 35 643 | 36 541 |  | 43 943 | 45 023 |
|  | 70-74 | 0.796 | 0.849 |  | 25.4 | 13.7 |  | 21 425 | 23 525 |  | 31 094 | 34 020 |  | 30 743 | 33 297 |
|  | 75-79 | 0.679 | 0.757 |  | 11.2 | 5.5 |  | 12 207 | 14 337 |  | 16 096 | 19 680 |  | 23 897 | 28 831 |
|  | 80+ | 0.568 | 0.624 |  | 11.2 | 5.5 |  | 10 587 | 16 031 |  | 12 511 | 18 985 |  | 16 333 | 24 919 |

Notes: NA: not available. Age-and sex-specific survival probabilities were from the 2015 China Life Tables from World Health Organization.^3^ The employment rates for the populations in 2010 in China were obtained from OECD.^4^ Estimated population numbers in the future were from the United Nation World Population Prospects.^5^

**Table S3 Annual direct medical，non-medical expenditure and work-loss days per liver cancer patient in China in 2019 by year post-diagnosis**

| **Characteristics** | | **No.** |  | **Direct medical expenditure** | | | | |  | **Direct non-medical expenditure** | | | | |  | **Work-loss days** | | | | |
| --- | --- | --- | --- | --- | --- | --- | --- | --- | --- | --- | --- | --- | --- | --- | --- | --- | --- | --- | --- | --- |
|  |  |  |  | **Year 1** | **Year 2** | **Year 3** | **Year 4-9** | **Year 10** |  | **Year 1** | **Year 2** | **Year 3** | **Year 4-9** | **Year 10** |  | **Year 1** | **Year 2** | **Year 3** | **Year 4-9** | **Year 10** |
| Overall | | 2139 |  | 62349 | 53892 | 43437 | 18168 | 91356 |  | 6096 | 4637 | 3461 | 1776 | 8931 |  | 58 | 52 | 41 | 17 | 85 |
| Age at diagnosis, years | |  |  |  |  |  |  |  |  |  |  |  |  |  |  |  |  |  |  |  |
|  | <45 | 332 |  | 59673 | 45728 | 34428 | 17388 | 87434 |  | 6669 | 3421 | 2175 | 1943 | 9772 |  | 57 | 41 | 35 | 17 | 84 |
|  | 45-59 | 989 |  | 66282 | 57910 | 30206 | 19314 | 97119 |  | 6358 | 5459 | 3469 | 1853 | 9315 |  | 63 | 61 | 37 | 18 | 93 |
|  | ≥60 | 818 |  | 58697 | 51085 | 60032 | 17104 | 86005 |  | 5524 | 3882 | 3838 | 1610 | 8094 |  | 52 | 42 | 47 | 15 | 76 |
| Gender | |  |  |  |  |  |  |  |  |  |  |  |  |  |  |  |  |  |  |  |
|  | Male | 1709 |  | 63816 | 54427 | 43390 | 18595 | 93505 |  | 6427 | 4676 | 3607 | 1873 | 9417 |  | 60 | 52 | 42 | 18 | 88 |
|  | Female | 430 |  | 55706 | 50530 | 44127 | 16232 | 81622 |  | 4595 | 4393 | 1310 | 1339 | 6732 |  | 48 | 51 | 26 | 14 | 71 |
| Region | |  |  |  |  |  |  |  |  |  |  |  |  |  |  |  |  |  |  |  |
|  | East | 988 |  | 61738 | 56399 | 48276 | 17990 | 90460 |  | 5833 | 4303 | 3766 | 1700 | 8547 |  | 57 | 56 | 43 | 17 | 83 |
|  | Central | 675 |  | 57475 | 39162 | 22926 | 16748 | 84214 |  | 7499 | 9653 | 1997 | 2185 | 10988 |  | 67 | 39 | 31 | 20 | 99 |
|  | West | 476 |  | 67486 | 52345 | 39884 | 19665 | 98883 |  | 5938 | 3623 | 3307 | 1730 | 8700 |  | 56 | 44 | 39 | 16 | 82 |
| Clinical stage | |  |  |  |  |  |  |  |  |  |  |  |  |  |  |  |  |  |  |  |
|  | I | 291 |  | 55888 | 41641 | 32094 | 16285 | 81889 |  | 5059 | 2771 | 2016 | 1474 | 7413 |  | 52 | 22 | 25 | 15 | 76 |
|  | II | 463 |  | 60014 | 53646 | 41832 | 17488 | 87935 |  | 5212 | 4329 | 3316 | 1519 | 7637 |  | 63 | 58 | 35 | 18 | 93 |
|  | III | 921 |  | 63156 | 52350 | 50397 | 18403 | 92539 |  | 6904 | 3401 | 3933 | 2012 | 10115 |  | 59 | 52 | 46 | 17 | 86 |
|  | IV | 368 |  | 63633 | 68841 | 44881 | 18542 | 93237 |  | 5779 | 9072 | 5152 | 1684 | 8468 |  | 56 | 59 | 50 | 16 | 81 |
| Health insurance | |  |  |  |  |  |  |  |  |  |  |  |  |  |  |  |  |  |  |  |
|  | UEBMI | 827 |  | 67416 | 55306 | 38632 | 19645 | 98781 |  | 6855 | 4393 | 3294 | 1998 | 10045 |  | 62 | 55 | 37 | 18 | 91 |
|  | URBMI | 381 |  | 63379 | 64018 | 43008 | 18468 | 92865 |  | 5723 | 4669 | 4709 | 1667 | 8385 |  | 50 | 61 | 49 | 15 | 73 |
|  | NRCMS | 851 |  | 58464 | 45587 | 51313 | 17036 | 85663 |  | 5639 | 4964 | 3052 | 1643 | 8262 |  | 59 | 44 | 43 | 17 | 86 |
|  | Commercial insurance | 19 |  | 29385 | 29385 | 29385 | 8563 | 43056 |  | 1062 | 1062 | 1062 | 309 | 1556 |  | 23 | 23 | 23 | 7 | 34 |
|  | Self-pay | 39 |  | 32613 | 130288 | 22183 | 9503 | 47786 |  | 3949 | 5397 | 2266 | 1151 | 5786 |  | 28 | 29 | 13 | 8 | 42 |
|  | Others | 22 |  | 65380 | 25384 | 25384 | 19051 | 95797 |  | 6595 | 1384 | 1384 | 1922 | 9663 |  | 67 | 18 | 18 | 19 | 98 |
| Pathological type | |  |  |  |  |  |  |  |  |  |  |  |  |  |  |  |  |  |  |  |
|  | Hepatocellular carcinoma | 1135 |  | 60857 | 51454 | 45077 | 17733 | 89169 |  | 6092 | 3663 | 2782 | 1775 | 8926 |  | 50 | 44 | 29 | 15 | 74 |
|  | Others | 228 |  | 57202 | 40941 | 40670 | 16668 | 83815 |  | 4967 | 10496 | 6202 | 1447 | 7277 |  | 58 | 52 | 83 | 17 | 86 |

Notes: All expenditure is expressed in CNY. UEBMI: urban employee basic medical insurance; URBMI: urban resident basic medical insurance; NRCMS: new rural cooperative medical system.

**References**

1. Institute for Health Metrics and Evaluation, University of Washington. GHDx. (2019). <http://ghdx.healthdata.org/gbd-results-tool>.
2. Lei H, Lei L, Shi J, et al. No expenditure difference among patients with liver cancer at stage I-IV: Findings from a multicenter cross-sectional study in China. *Chin J Cancer Res*. 2020;32(4):516-529.
3. World Health Organization. Life tables for China in 2015. <http://apps.who.int/gho/data/view.main.60340?lang=en>.
4. Organization for economic co-operation and development. Employment rate. (2019). <https://data.oecd.org/gdp/gdp-long-term-forecast.htm>.
5. United Nations. Population Division. World Population Prospects 2019. https://population.un.org/wpp/.
